# Identification of a novel, dominant dwarfing gene (*Ddw4*) and its effect on morphological traits of rye

**DOI:** 10.1371/journal.pone.0199335

**Published:** 2018-06-18

**Authors:** Zuzanna Kantarek, Piotr Masojć, Anna Bienias, Paweł Milczarski

**Affiliations:** Department of Genetics, Plant Breeding and Biotechnology, West Pomeranian University of Technology, Szczecin, Słowackiego 17, Poland; Institute of Genetics and Developmental Biology Chinese Academy of Sciences, CHINA

## Abstract

Shortening rye stems to improve lodging resistance is among the major tasks awaiting breeders of this cereal. The most straightforward way to achieve this goal is the implementation of a dominant dwarfing gene into high yielding cultivars. The choice of dominant dwarfing genes in rye is limited to *Ddw1* and *Ddw3* loci, which are well characterized with respect to map position and tightly linked molecular markers on the long arms of chromosomes 5RL and 1RL, respectively. This paper reports on the identification and preliminary characterization of a novel dominant dwarfing gene, *Ddw4*, from line S44. This was mapped within the centromeric region of chromosome 3R. The *Ddw4* gene is sensitive to exogenous gibberellin. Its introduction into the rye populational cultivar Dańkowskie Amber decreased plant height by c. 54% without any negative effects on spike length and number of kernels per spike. Further genetic studies are needed to determine the perspectives for application of the newly detected dwarfing gene into breeding programs for short-stem rye.

## Introduction

In the majority of the studied mapping populations, plant height shows quantitative variation controlled by complex networks of interacting quantitative loci (QTL), as revealed in rice [1], wheat [2], barley [3–4], and rye [5–7]. Because of the high number and small and/or unstable effects, QTL are usually ineffective tools for practical breeding. More interesting for practical purposes are rare monogenic mutations causing radical growth reduction that have been identified among genetic stocks. Dominant dwarfing genes-*Rht-B1b (Rht1)*, *Rht-D1b* (*Rht2*) (located on short arms of chromosomes 4B and 4D), and *Rht8* (2DS) have been introduced into wheat cultivars to reduce plant height [8–9]. A similar strategy for breeding short-straw cultivars of barley is based on the semidwarf gene *denso* (*sdw1*) located on chromosome 3HL [10–11]. Semidwarf *sd-1* gene from chromosome 1, introduced in the course of “green revolution” into rice cultivars, is a defective GA20 oxidase allele [12]. Recently, *sdw1* and *sd-1* semidwarf genes were established as being orthologous [13]. Rye cultivars still represent a tall-straw phenotype, which makes them sensitive to lodging. So far, attempts to identify dominant dwarfing genes in this species have resulted in the characterization of the *Ddw1* gene on chromosome 5RL [14–15], the *Ddw2* gene on chromosome 7R [16–17] and the *Ddw3* gene on chromosome 1RL [18]. Only *Ddw1* and *Ddw3* genes are more closely characterized and tagged by molecular markers. This paper reports on the identification of a new dominant dwarfing gene, *Ddw4*, in rye and presents its basic characteristics in view of its possible application in breeding.

## Materials and methods

### Plant material

A dwarf line was selected from within the S44 population of rye (*Secale cereale* L.) lodged in the National Center for Plant Genetic Resources: Polish Genebank IHAR, accession number 31147. The population was internally differentiated in respect to plant height and contained high, medium and short plants. Plants of c. 80 cm height were self-pollinated in two consecutive years, which led to the development of a dwarf inbred line denoted line S44. A second line, 541, representing tall plants, originated from the Department of Genetics, Plant Breeding and Biotechnology, West Pomeranian University of Technology in Szczecin, Poland [19]. Dwarf line S44 is 2 days earlier than tall line 541 in respect to heading date and flowering time, while both lines are 5–7 days later than cv. Dańkowskie Amber. The F_1_ (541 × S44) cross was developed in 2012. The F_2_ progeny was obtained through self-pollination of F_1_ plants. Each F_2_ plant grown in 2013 was self-pollinated giving seeds of F_3_ progeny. Recombinant inbred lines (RILs) were further developed using the single seed descent (SSD) method. F_2_ plants were phenotyped as short or tall by visual observation and height measurement. F_2_ plant phenotype was further confirmed by observation of the F_3_ progeny (7–20 plants per line). The hypothesis of the monogenic inheritance of plant height in F_2_ and F_3_ progenies was verified using the χ^2^ test. F_2_ plants were also analyzed in respect to: length and thickness of the second internode from the base, spike length, grain number and weight per spike. In order to obtain a short-straw version of the highly yielding cultivar Dańkowskie Amber, and F_1_ progeny of the cross Dańkowskie Amber × S44, was developed. A preliminary characterization of these newly developed plant materials was conducted in respect to morphological traits, including thousand-kernel weight (TKW). All field experiments were performed at the Experimental Station of the West Pomeranian University of Technology in Szczecin, Poland. The significance of the differences between genotypes was tested using a t-student test. Correlation coefficient was calculated on average data. Computations were performed using the Statistica 13.1 software (Dell Inc.).

### Genetic mapping

The F_2_ mapping population for the 541 × S44 cross consisted of 87 lines, including 64 short-straw and 23 tall plants drawn from the entire population of 519 F_2_ plants based on phenotypic performance within F_3_ progenies consisting of at least 15 mature plants. DNA was isolated from each of the 89 F_2_ and parental lines in early spring using a DNeasy Plant Mini Kit (Sigma), according to the protocol delivered by the manufacturer. Genotyping by sequencing (GBS) was performed using DArTseq technology (Diversity Arrays Technology Pty Ltd, Australia), according to Li *et al*. [20]. Markers generated “*in silico*” and in single nucleotide polymorphisms (SNP) versions were taken for constructing linkage groups. DArTseq markers were assigned to rye chromosomes by applying the reference map of the 541 × 2020 cross [21]. Mapping of the dwarfing gene was conducted as the final step of map construction. A computer package, JoinMap 5.0 [22], was used for linkage analysis. Linked markers were grouped at LOD = 18. An original map drawing the relationships to the reference map was generated using MapChart 2.2 software [23].

### Test for sensitivity to exogenous GA_3_

Seedlings of the two parental lines 541 and S44 were subjected to exogenous GA_3_ treatment according to Worland [8]. Kernels distributed on a water paper soaked with distilled water in a Petri dish (φ = 12 cm) were kept for 48 hours at 4°C to synchronize their germination. Each line was represented by 10 germinating seeds in 3 replications for distilled water (control) and 5 ppm GA_3_ treatments. The lengths of seedlings grown for 14 days (2–3 leaves stage) were measured and the ratio between GA_3_ treated and water treated plantlets was determined. A non-parametric Kolmogorov-Smirnov test was used to assess the significance of the reaction to GA_3_ (Statistica 13.1).

## Results

### Genetic analysis of plant height in 541 × S44 crosses

**Fig 1:**
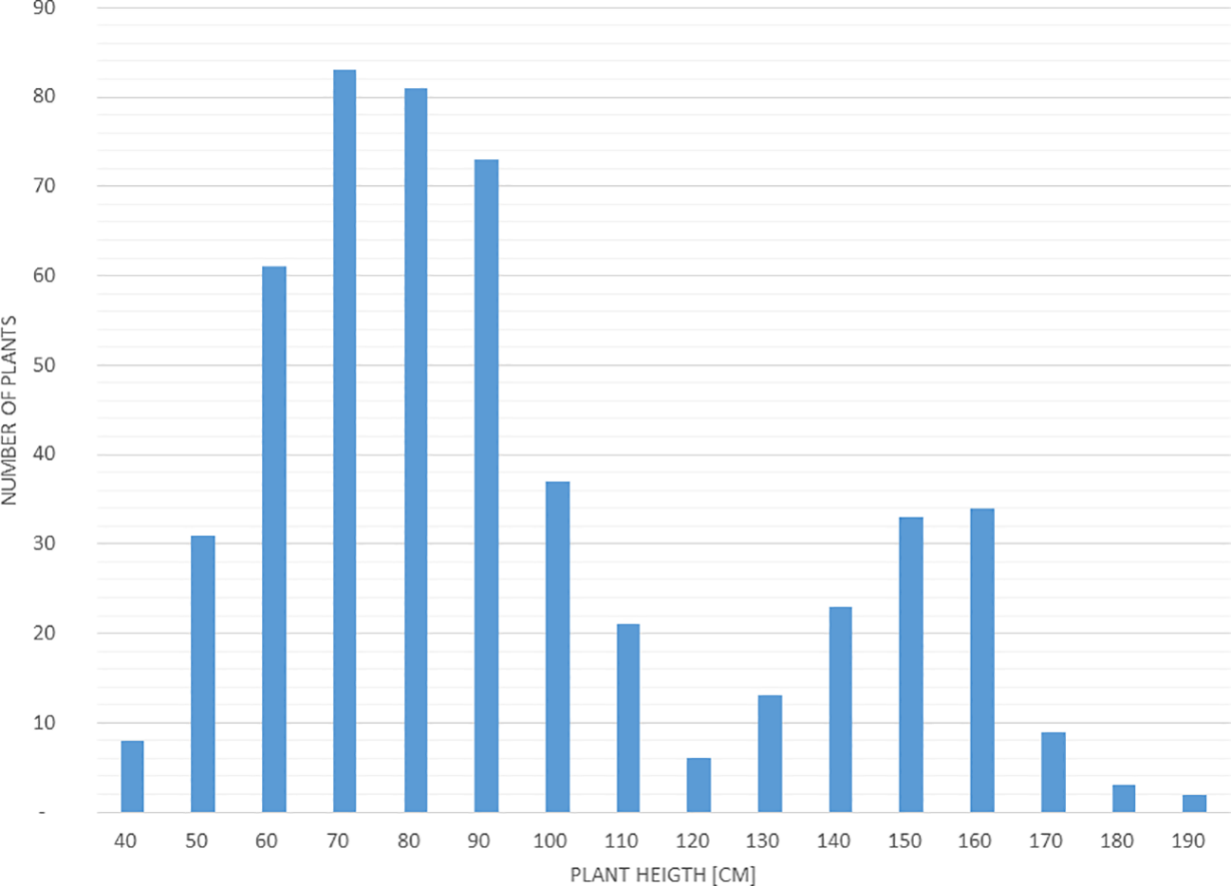
Distribution of plant height in F_2_ generation of the 541 × S44 cross.

The stability of line S44 across three years in respect to the short-straw phenotype is shown in Table 1. The mean height of this line ranged from 71.3 cm in 2015 to 84.8 cm in 2014. The three-year mean height was 77.2 cm, which constituted 57% of that found for line 541 grown in the same environment. The height of the F_1_ generation was 85 cm and this result suggested complete dominance of the dwarfing gene over the respective allele from line 541. The range of plant height (40.0–190.5 cm) found in the F_2_ generation consisting of 518 plants (Fig 1) exceeded that of parental lines, which suggests environmental or genetic background effects, including transgression. The plant height distribution in F_2_ progeny was bimodal, showing that this generation consists of two subpopulations: a more numerous short-straw one (40–120 cm) with a mean value of 81.6 cm; and a minor one, representing tall plants (130–190 cm) with a mean value of 156 cm. The lowest height attributed to tall plant phenotypes was 130 cm, since from this value no dwarf plants were observed in F_3_ progeny. Analysis of F_3_ progenies allowed the differentiation of homozygotes from heterozygotes within the dwarfing gene locus. The average height of dwarf homozygotes in the F_2_ generation was 72.8 cm, and that for heterozygotes was 87.7 cm, which suggests a high degree of dominance for the dwarfing gene. The segregation ratio of short-straw vs. tall phenotypes in F_2_ confirmed by observation of F_3_ progenies was consistent with the 3:1 ratio, suggesting the monogenic inheritance of plant height in the 541 × S44 cross (Table 2).

**Table 1:** Characteristics of morphological traits in parental lines (541 and S44) for mapping population.

Year		Plant height* [cm]		Length of 2-nd internode* [cm]		Thickness of 2-nd internode [mm][		Spike length* [cm]		Number of kernels per spike		Weight of kernels per spike	
		541	S44	541	S44	541	S44	541	S44	541	S44	541	S44
2014	Mean	137.15	84.80	19.40	14.80	4.53	4.22	11.24	9.98	49.90	57.90	1.15	1.41
	SD	4.63	10.53	1.61	3.47	0.32	0.30	0.68	1.55	7.05	6.76	0.26	0.28
2015	Mean	135.20	71.30	18.80	14.60	3.88	3.91	11.99	9.36	50.60	36.60	1.16	0.91
	SD	9.94	6.20	1.47	3.16	0.40	0.69	1.15	0.98	6.55	14.21	0.18	0.41
2016	Mean	132.90	75.50	19.26	13.42	4.68	4.63	10.70	10.72	54.20	46.10	1.74	1.23
	SD	5.80	8.61	3.59	1.93	0.58	0.68	0.69	1.93	15.14	14.82	0.71	0.47
**2014 2016**	**Mean**	**135.08**	**77.20**	**19.15**	**14.27**	**4.36**	**4.25**	**11.31**	**10.02**	**51.57**	**46.87**	**1.35**	**1.18**
	**SD**	**6.79**	**8.45**	**2.22**	**2.85**	**0.43**	**0.56**	**0.84**	**1.49**	**9.58**	**11.93**	**0.38**	**0.39**

*- traits showing significant differences between parental lines, t–Student test at p<0.0001

**Table 2:** Segregation of dwarf vs. tall phenotypes among plants of F_2_ progeny, supported by analysis of F_3_ generation.

Generation	Range of plant height [cm]	Dominant homozygotes	Heterozygotes	Reccessive homozygotes	Expected segregation ratio	χ^2^
F_2_	40.0–190.5	386		131	3: 1	0.052 (n.s.)*
F_3_	40.0–187.0	125	251	121	1: 2: 1	2.38 (n.s.)

*-n.s.- not significant deviation from the Mendelian ratio as assessed by χ^2^ test at p<0.05

### Mapping of the dwarfing *Ddw4* gene in rye

**Fig 2:**
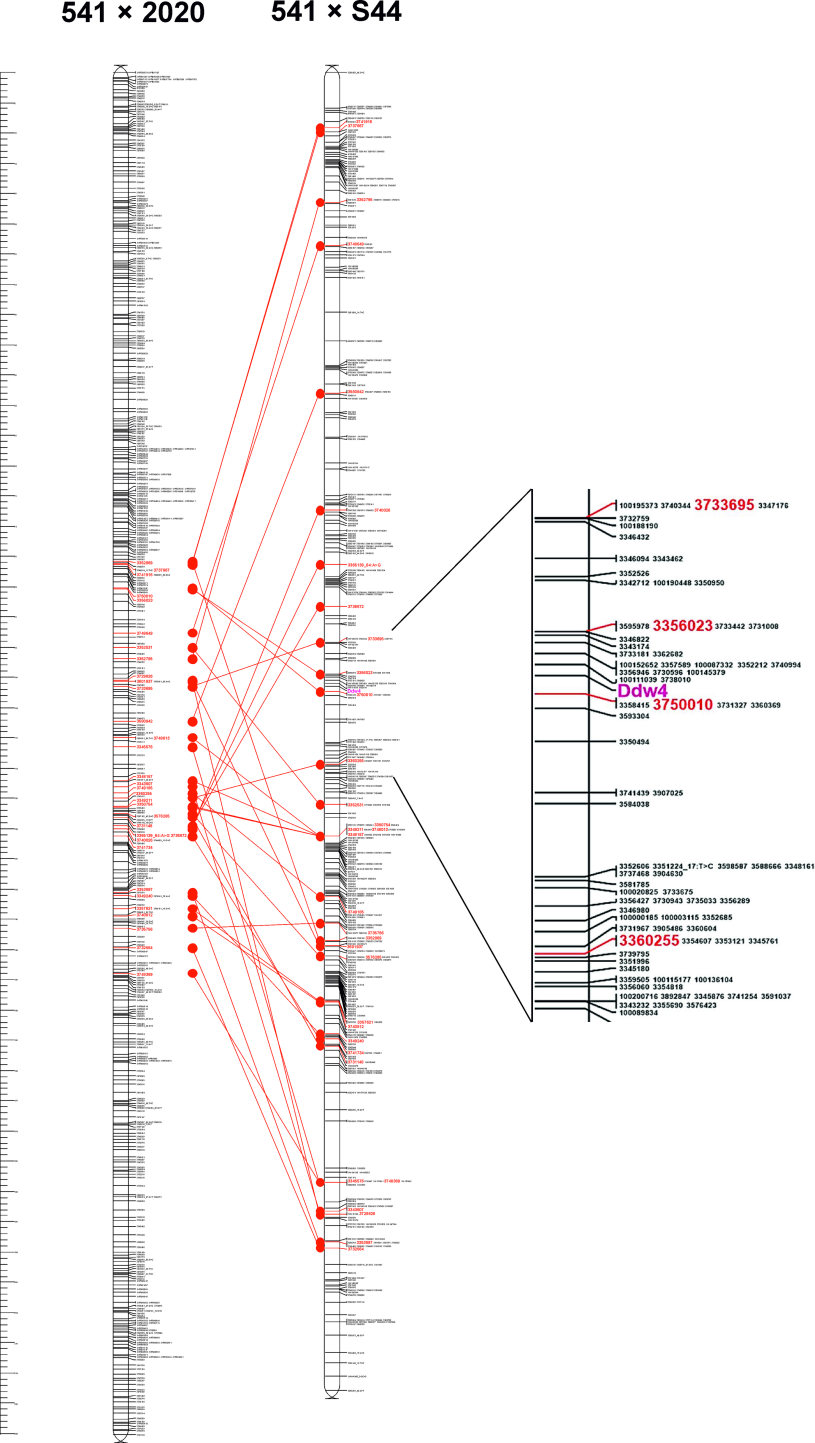
Chromosomal localisation of *Ddw4* gene. The reference map of 3R chromosome (541 × 2020) comes from Milczarski *et*.*al*. [21].

Out of 77 573 DArTseq markers, 17 317 were found to segregate in a Mendelian mode (3:1 segregation ratio) within the F_2_ mapping population of the 541 × S44 cross. Linkage groups relevant to particular rye chromosomes contained 1910 (1R), 1602 (2R), 1797 (3R), 2973 (4R), 1578 (5R), 1536 (6R) and 1673 (7R) markers. The dwarfing gene was mapped on the long arm of chromosome 3R within the centromeric region (Fig 2, S1 Table). The high-density mapping of *Ddw4* was achieved using 595 markers located within a distance of c. 70 cM to the gene locus, and these were collected with the help of a chromosome 3R reference map. The gene is located within a cluster of 18 markers (linkage < 1 cM) which might be used for its tagging and performing marker assisted selection. Since the map position of the dwarfing gene is here reported for the first time, the novel gene was assigned the symbol *Ddw4* showing that it is the fourth dominant dwarfing gene found in rye.

### Sensitivity of *Ddw4* to GA_3_ treatment

**Fig 3:**
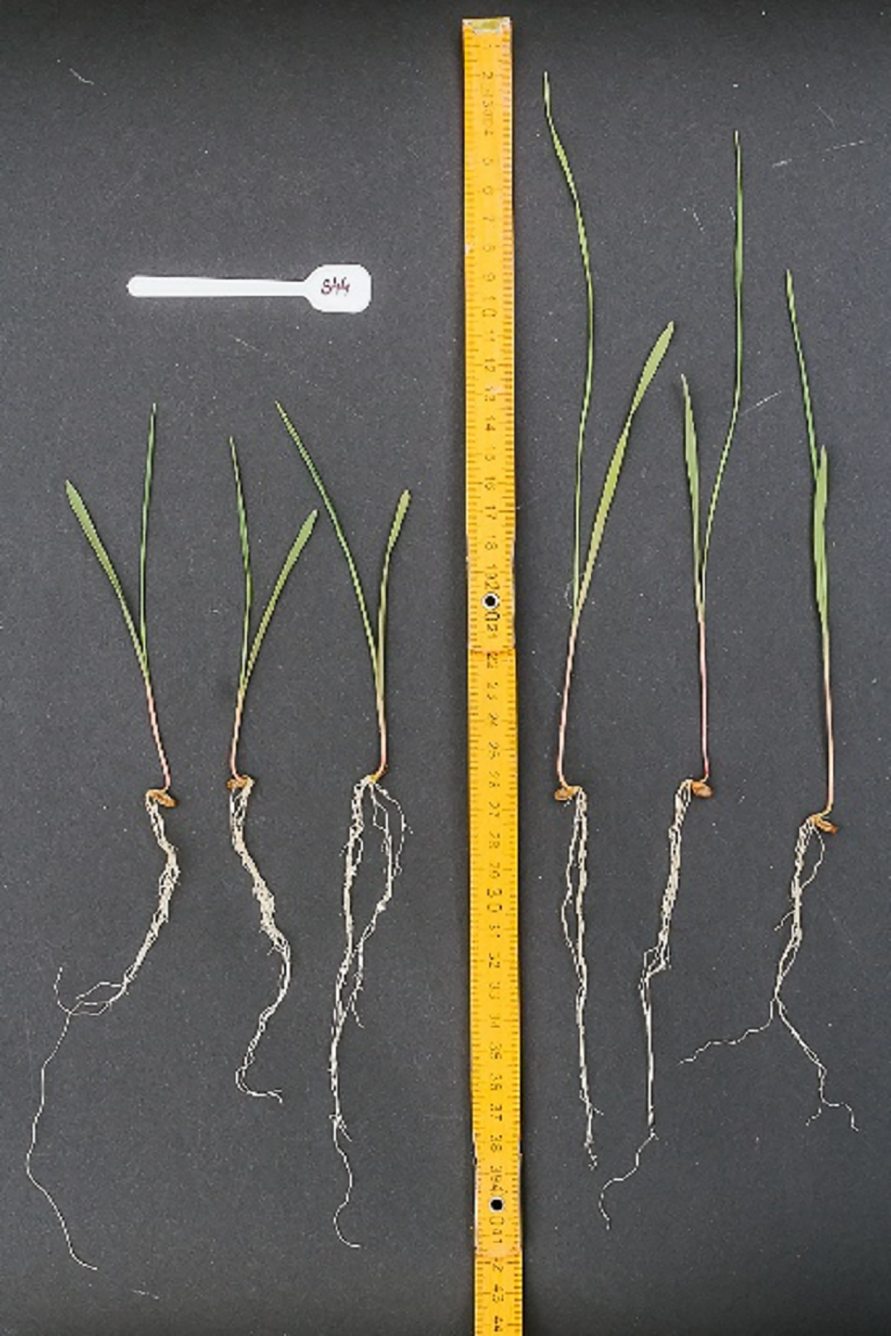
Reaction seedlings of line S44 to treatment by GA_3_. The seedlings on the left side of the ruler were treated with water and on the right side with GA_3_ solution.

The S44 line showed a high degree of sensitivity to exogenous GA_3_ at the seedling stage (Table 3, Fig 3). The response (177% increase in seedling length) was much higher than that shown by line 541 expected to be highly sensitive to GA_3_. Thus, it may be concluded that the *Ddw4* gene belongs to the group of GA_3_ sensitive dwarfing genes.

**Table 3:** Response of parental lines for the 541×S44 cross on GA_3_ treatment.

Genotype	Mean length of seedlings [cm]		GA_3_/control	Level of significance
	control (H_2_O)	GA_3_ treated		
541	16.53	23.80	144.4%	p < 0.05
S44	11.35	20.11	177.2%	p < 0.05

### Assessment of *Ddw4* effects on other agronomic traits of rye

The conspicuous difference between parental lines S44 and 541 in respect to plant height is associated with the significant difference between the second internode and spike length (Table 1). However, three other traits of agronomic value, i.e. the 2nd internode thickness, kernel number and kernel weight per spike, seem to be unrelated to plant height. Positive significant correlation coefficients between plant height and kernel number per spike (r = 0.32), plant height and kernel weight per spike (r = 0.56) as well as those between kernel number per spike and kernel weight per spike (r = 0.72) found in the F_2_ progeny suggest that these two traits might be negatively affected by the *Ddw4* dwarfing gene (Table 4). On the other hand, finding that plant height and spike length are not correlated within the F_2_ progeny may provide positive perspectives for application of *Ddw4* gene in breeding.

**Table 4:** Significant correlation between analyzed traits within mapping population 541 × S44 (p < 0.05).

	Plant height	Length of 2-nd internode	Thickness of 2-nd internode	Spike length	Number of kernels per spike	Weight of kernels per spike
Plant height	-					
Length of 2-nd internode	**0.51**	-				
Thickness of 2-nd internode	**0.14**	**0.12**	-			
Spike length				-		
Number of kernels per spike	**0.32**	**0.27**	**0.21**	**0.10**	-	
Weight of kernels per spike	**0.56**	**0.43**	**0.25**	**0.13**	**0.72**	**-**

Preliminary results showing *Ddw4* effects on agronomic values of cv. Dańkowskie Amber are shown in Table 5. The dwarfing gene is apparently dominant in the F_1_ generation as it reduces the height of cv. Dańkowskie Amber by c. 54%. This strong positive effect on plant height is not associated with negative effects on thickness of the second internode, spike length, kernel number or kernel weight per spike. Unfortunately, the *Ddw4* gene negatively affects the thousand-kernel weight of this cultivar by c. 31%.

**Table 5:** Characteristics of morphological traits in cv. Dańkowskie Amber and its F_1_ cross with inbred line S44.

	Plant height* [cm]		Length of 2-nd internode* [cm]		Thickness of 2-nd internode [mm[		Spike length [cm]		Number of kernels per spike		Weight of kernels per spike [g]		TKW* [g]	
	Mean	SD	Mean	SD	Mean	SD	Mean	SD	Mean	SD	Mean	SD	Mean	SD
Dańkowskie Amber	132.10	7.21	12.00	1.18	3.80	0.03	10.12	0.91	43.10	7.89	1.87	0.54	33.63	4.03
Dańkowskie Amber x S44 (F_1_)	74.55	7.4	8.85	1.42	3.40	0.70	10.20	0.75	50.10	5.20	1.61	0.21	23.52	2.65

*- traits showing significant difference between Dańkowskie Amber and F_1_ (Dańkowskie Amber × S44), t–Student test at p<0.001

## Discussion

Dominant dwarfing genes have not previously been reported on chromosome 3R. There is one early paper by De Vries and Sybenga [24] attributing recessive dwarfing gene *dw3* to this chromosome [17]. Since the map position of *dw3* was not established it is difficult to decide on its relationship to *Ddw4*. The newly reported *Ddw4* gene was precisely localized on the high-density molecular map of chromosome 3R in the centromeric region on the side of 3RL chromosome arm. This position corresponds to major QTL of the D class for plant height, revealed by Masojć *et al*. [7] using a 541 × Ot1–3 mapping population of rye and a high-density map of DArT markers. The latter study shows that the height of the 541 line is controlled by a complex system of interacting QTL distributed on each of rye chromosomes, which means that the *Ddw4* mutation should represent a defective allele in a key locus. Because of the complexity of QTL systems for plant height in rye, some background effects caused by other possible QTL within the 541 × S44 cross might be expected and indeed they can explain a wide range of the variation found in the F_2_ generation. A centromeric region on barley chromosome 3HL has been found to contain the recessive dwarfing gene *uzu* [25]. Due to the high level of synteny between 3H and 3R chromosomes, *Ddw4* is possibly orthologous to its counterpart from barley.

The application of *Ddw4* in breeding short-straw, lodging resistant rye varieties seems promising in view of its performance in the F_1_ cross between cv. Dańkowskie Amber and S44 line. The achieved short-stature of rye plants without shortening of spikes is satisfying. However, the significant reduction in thousand-kernel weight observed in this cross needs further explanation. This negative effect might result from the genetic background inferred by line S44 or by *Ddw4* gene itself. Backcrossing with cultivar components and selection for dwarfing alleles should clarify this problem. The chromosome 3RL region containing the *Ddw4* locus is also a QTL localization place for thousand-kernel weight, kernel length, kernel thickness and kernel number per spike [5]. Close linkage between these QTL and the dwarfing gene may result in association of grain parameters with plant height, which means that allele compositions in these loci in a short-straw donor line is crucial for the agronomic performance of developed progenies. The considered chromosome 3R region was shown to contain QTL controlling benzoxazinoid content and leaf rust resistance [26] which in turn raises the question about the response of *Ddw4* to brassinosteroids and its association with disease resistance. *Ddw4* may also be associated with preharvest sprouting resistance (PHS) and alpha-amylase activity in the grain (AA), as it is tightly linked with QTL for these traits [27–30] and with GA_3_ oxidase [31], a candidate gene for PHS.

The strong response from the S44 line to exogenous GA_3_ positions the *Ddw4* gene in the same group of GA-sensitive dwarfing genes as *Ddw1* and *Ddw3* [17–18]. Therefore, it can be concluded that *Ddw4* does not represent GA signaling genes. Neither of these dwarfing genes is an ideal candidate for implementation of a short-stem phenotype into rye cultivars due to the observed negative effects on yield-related traits underlined both by Stojałowski *et al*. [18] and in this study. So far, attempts to reduce plant height in rye have been made with the use of the *Ddw1* gene, especially in Finland [15]. With three dominant dwarfing genes identified and to be tagged by molecular markers in the near future, a wide-ranging search for the most valuable genetic backgrounds eliminating undesirable effects should be initiated.

## Supporting information

S1 Table Markers segregation in mapping population 541 x S44 on chromosome 3R. (XLSX)
